# A magnetically recyclable photocatalyst with commendable dye degradation activity at ambient conditions

**DOI:** 10.1038/s41598-018-32911-3

**Published:** 2018-10-02

**Authors:** Abhilasha Pant, Ruchika Tanwar, Bikramjit Kaur, Uttam Kumar Mandal

**Affiliations:** University School of Chemical Technology, G.G.S. Indraprastha University, Sector 16C, Dwarka, New Delhi 110078 India

## Abstract

An efficient, economical, environment-friendly and easy separable catalyst to treat environmental contaminants is an enduring attention in recent years due to their great potential for environmental protection and remediation. Here we have reported the excellent performance of polyaniline activated heterojunctured Ni_0.5_Zn_0.5_Fe_2_O_4_ catalyst to degrade azo dye in an aqueous solution at ambient condition. The catalyst was prepared via a simple facile polymerization procedure. The physicochemical properties and structure of the synthesized catalyst was confirmed by TGA, PXRD, FTIR, SEM, HRTEM, XPS, EDX, and DRS techniques. The developed catalyst has shown an accelerated degradation ability of an organic pollutant Orange ll Sodium salt azo dye about 100% for the dye concentration of 50 ppm within five minutes at ambient conditions with 1 g/l loading of catalyst. Simple facile synthesis, easy separation by an external magnet, good reusability and high degradation capability of the catalyst may promote the practical applications of the heterostructured catalyst at ambient condition for water remediation. The present study also explored possible credible charge transfer directions and mechanism of photocatalysis supported by trapping experiments and electrochemical impedance spectroscopy (EIS) measurement for the effective improvement of photocatalytic activity and enhancement of the visible light adsorption.

## Introduction

A wide range of applications of synthetic dyes in various industries like food, textile, printing, leather and other industries and rapid increase in the production of dye effluent has become one of the major contributors to water pollution and strongly impacted on the balancing of nature^1^. The release of huge amounts of synthetic dyes in the effluents is a thriving challenge to sustain environmental protection and a slight concentration of dye as low as 10 ppm affects the transparency of water, lowers the gas or oxygen dissolving capacity in water and adversely impacts on the aquatic life due to reduction in photosynthesis, besides, some of the dyes possess carcinogenic and mutagenic affects^2–4^. Several treatment methods and technologies have been designed and developed, including Fenton like reactions, adsorption techniques, reductive degradations using zero-valent iron, biological degradations, photocatalysis for the removal of synthetic dyes from the aqueous effluents to reduce their concern on the environment^5,6^. Among these processes, owning to milestone research, semiconductor photocatalyst, as a green catalyst, has garnered major acclaim for the purifying organic pollutants present in water and has become as an effective and potential solution to degrade synthetic dyes like azo based dye for environmental sustainability^7^. Although it is believed that TiO_2_ as one of the most important applied workhorse heterogeneous photocatalyst degrades azo dyes thoroughly with unrivalled efficiency but it cannot absorb sun light and possesses impeded efficiency and restricted in utilization as a visible light photocatalyst due to its wide band gap (e_g_ = 3.2 eV for anatase)^8^. To alleviate this limitation many reports have been appeared and directed reinforcement in the catalytic performance of the photocatalyst under visible light/ambient conditions^9,10^. Towards this goal, the developed quantised technologies are still under thriving strategies and limits it’s practical applications. Therefore, there is great interest to design catalysts for dye degradation without complicacy of light illumination as in the field of photocatalysis as well as additional reagents like H_2_O_2_/O_3_ in Fenton like catalytic degradation.

With this particular interest, lately, several groups have integrated the semiconducting photocatalysts with delocalised conjugated compounds/polymers such as graphene, carbon nanotubes, polyaniline, polypyrorole etc into a single nanoscale heterostructure to exploit the novel strides as sensitizers by creating high extinction coefficient and wide spectral range as well as on tuning band gaps for harvesting low energy photons^11–13^. Among these, the integration with the delocalised conjugated polyaniline (PANi) has been received a potential platform to tailor light absorption owing to its good conductivity, easy facile synthesis, reversible acid-base forming ability and exceptional environmental stability^14^. In the last two decades, the significant photocatalysis performance has been shown in visible light range by PANi due to presence of an extended π-conjugated electron and easy movement of charge carriers. Many mechanistic approaches have also been established to explain the role of PANi as a benign sensitizer for semiconductor photocatalysts and enhancement in catalytic performance^15^. The additional prospect of the remarkable catalytic performance by the integration of semiconductor-PANi has also been accepted by the researchers that PANi not only acts as a good electron donor from its LUMO orbital but also as an excellent hole acceptor/conductor in LUMO orbital after harvesting in visible light and substantially retards the recombination rate of light induced charge-hole carriers^16^. It is established that the low energy excitation of PANi through π-π* transition offers a potential platform for designing the semiconductor based photocatalysts with complete suppression of photocorrosion and enhanced catalytic activity^17^.

Recently, besides the potential utilizations of heterostructured PANi-semiconductor photocatalysts, another approach is adopted by researchers to recover and reuse of nanoscale photocatalysts after degradation reaction in slurry for sustainable process management through introduction of magnetic nanoparticles in solid matrices for easy handling of the catalysts^18,19^. It is reported that the magnetic photocatalysts have not only showed high catalytic activity under UV/visible light but also facilitate the easy separation by an external magnetic field from aqueous solution after degradation. Therefore from the above understanding and with inner great interest we planned to study the Ni_0.5_Zn_0.5_Fe_2_O_4_@PANi hybrid metal oxides nanocomposites for the fascination that may not only accomplish the attainment of some exceptional catalytic performance but can also be utilised as a simple cost-effective magnetic separation technology.

For the easy separation and reduction in photocorrosion it has been reported recently that many magnetically separable hybrid nanocomposites like hybridized PANi-magnetic nanoparticles have showed potential photocatalytic degradation of organic pollutants in aqueous phase under visible light^15,19,20^. However, this heterostructured hybrid catalyst has distinct features like excellent photocatalytic degradation under the visible light, complete recovery, reusability and resistant to photocorrosion. For the sake of bright fringes in the photon activated photocatalytic performance by these hybrid catalysts it is interesting to design photocatalysts which could be very efficient and could absorb the incident light at ambient conditions effectively as much as possible. Among magnetic nanoparticles, NiFe_2_O_4_/CoFe_2_O_4_ based hybrid nanocomposites with PANI have comparatively better catalytic activity and can be easily separated by external magnet^21,22^. On the other hand introducing Zn^2+^ into NiFe_2_O_4_ could inhibited phase transition and assure better magnetic properties of Ni_0.5_Zn_0.5_Fe_2_O_4_. On hybridization of Ni_0.5_Zn_0.5_Fe_2_O_4_ with PANi, an interface may be formed not only to harvest visible light efficiently but also positively facilitates catalyst separation and suppose to be creates an induced synergistic role for degradation of organics with an exceptional activity^23^. In the present study, we have used specific molar composition of Ni_0.5_Zn_0.5_Fe_2_O_4_ as this is a well-established inverse spinal hybrid nanocrystal with general formula Zn_A_^2+^Fe_A_^3+^[Ni_B_^2+^Fe_B_^3+^]O_4_. The cations within the brackets are located at octahedral (B) sites and Zn incorporation with mole composition i.e., ~50% reduces the number of Fe ions on A(tetrahedral) sites and weaken interaction between the A and B sublattices of the nanocrystal and as a result magnetic moments of Fe arrange collinearly showing highest magnetic saturation^24,25^. Also here we have demonstrated a very simple solvothermal process and easy straightforward strategy to prepare magnetically separable Ni_0.5_Zn_0.5_Fe_2_O_4_@PANi photocatalyst via an *in-situ* oxidative polymerization and its catalytic activity for removal of Orange II sodium salt, a non-biodegradable anionic azo dye, from water under ambient conditions. Interestingly, we found that the Ni_0.5_Zn_0.5_Fe_2_O_4_@PANi photocatalyst with 1:1 by weight ratio shows exceptional catalytic degradation to azo dye under ambient conditions without additional light sources or additional reagents.

## Results

**Figure 1:**
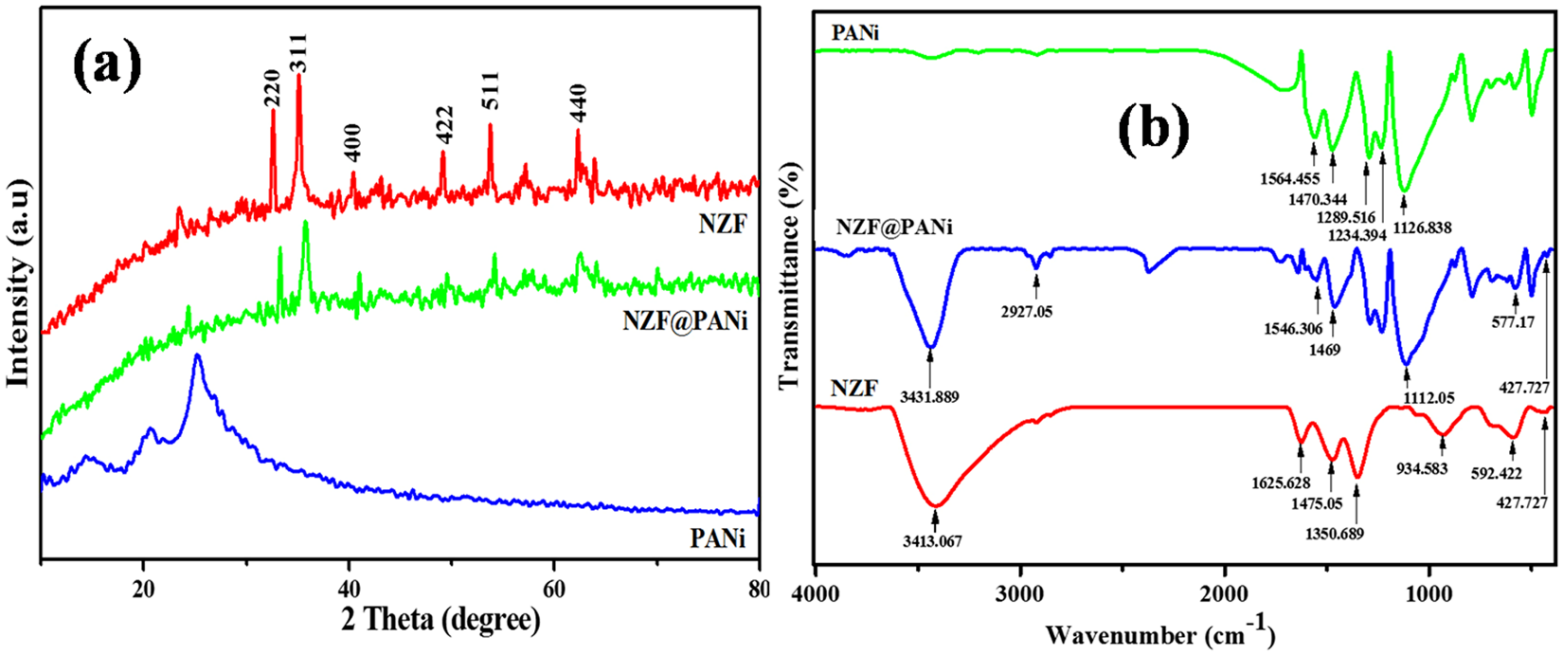
XRD (**a**) and FTIR (**b**) pattern of NZF, NZF@PANi and PANi.

The X-ray diffraction patterns (XRD) of NZF and NZF@PANi are presented in Fig. 1a. The diffraction peaks of NZF nanocrystals at 2θ = 30.01°, 35.23°, 42.50°, 52.82°, 56.74° and 62.20° correspond to the reflections of (220), (311), (222), (400), (422), (511) and (440) planes, respectively and are indexed to the spinel structure of Ni_0.5_Zn_0.5_Fe_2_O_4_ (a = 8.346 Å, JCPDS No. 08-0234)^26^. The broad diffraction peaks focused at 2θ = 16, 20 and 25° are attributed to the semicrystalline phases of the HCl doped polyaniline chains periodicity^26^. The XRD pattern of the NZF@PANi nanofibers revealed that diffraction peaks correspond to the polyaniline semicrystallinity disappeared due the restricted polymer chains alignment in presence of nanopartciles^27^ and the crystalline peaks correspond to NZF nanocrystals are focused at (311), (222), (400), (422), (511) with reduced intensity due to polymer coating. The surface attachment/coating of PANi on NZF nanoparticles did not affect the spinal-type crystal structure. The average crystallite sizes of the pure NZF nanocrystals and hybrid composite particles were estimated by using Debye-Scherrer equation:

1 $${\rm{D}}={\rm{k}}{\rm{\lambda }}/{\rm{\beta }}\,\cos \,{\rm{\theta }}$$

here, D is the crystallite size, k is a dimensionless factor, which is taken as 0.9, β is the full width at half maximum of the most intense diffraction peak of 311 planes for NZF nanocrystals and NZF@PANi, respectively. λ is the wavelength of the Cu target (1.5406 Å), and θ is the Bragg diffraction angle. The d values, lattice constants and the average crystallite size of the pure NZF and NZF@PANi composite are summarized in Table S1 (Supplementary Information).

The FTIR spectra were recorded to outline the molecular fingerprints of the nanocomposite. The Fig. 1(b) shows a comparative analysis of the three FTIR spectra of PANi, NZF@PANi and NZF respectively. From the FTIR spectrum for NZF, the bare magnetic nanoparticles exhibit a broad peak between 3500–3350 cm^−1^ is due to –OH stretch band which gets associated with the lattice surface of ferrite at the time of sample preparation by co-precipitation method^28^. The band appears at 1625 cm^−1^ is attributed to the stretching vibration of the hydrogen bonded O-H groups and the bands at 1550–450 cm^−1^ and 960-875 cm^−1^ are attributed to the in-plane and out-plan -OH bonds respectively^29^. The two broad bands at 592 cm^−1^ and 500-430 cm^−1^ are corresponding to intrinsic stretching vibrations of the metal at the tetrahedral and octahedral sites. The characteristic FTIR spectrum of PANi is shown in Fig. 1(b). It is clearly seen that main characteristic bands of PANi are 1564 cm^−1^ and 1470 cm^−1^ correspond to the quinonoid and benzenoid rings and clearly unveil the presence of emeraldine form of PANi and the peaks at 1289 cm^−1^ and 1232 cm^−1^ can be attributed to the C-N bond stretching mode of benzene ring. The very strong and wide peak at 1120 cm^−1^ is delineated as the “electronic-like band” of quinoid unit of doped PANi^30^ The characteristics adsorption peaks of both PANi and NZF can be found in the NZF@PANi composite spectrum. However the characteristic peaks of PANi in the NZF@PANi composite shift to lower wave number as compared to pure PANi and found to be at 1550 cm^−1^, 1461 cm^−1^ and 1112 cm^−1^. These shifts of the characteristic peaks can be attributed to the interaction between quinone and quinonoid nitrogen of PANi polymer chains and metal-oxide linkages of NZF which delocalised the electron density and bond energy of the PANi after the incorporation of NZF during *in-situ* polymerization^26,31^.

**Figure 2:**
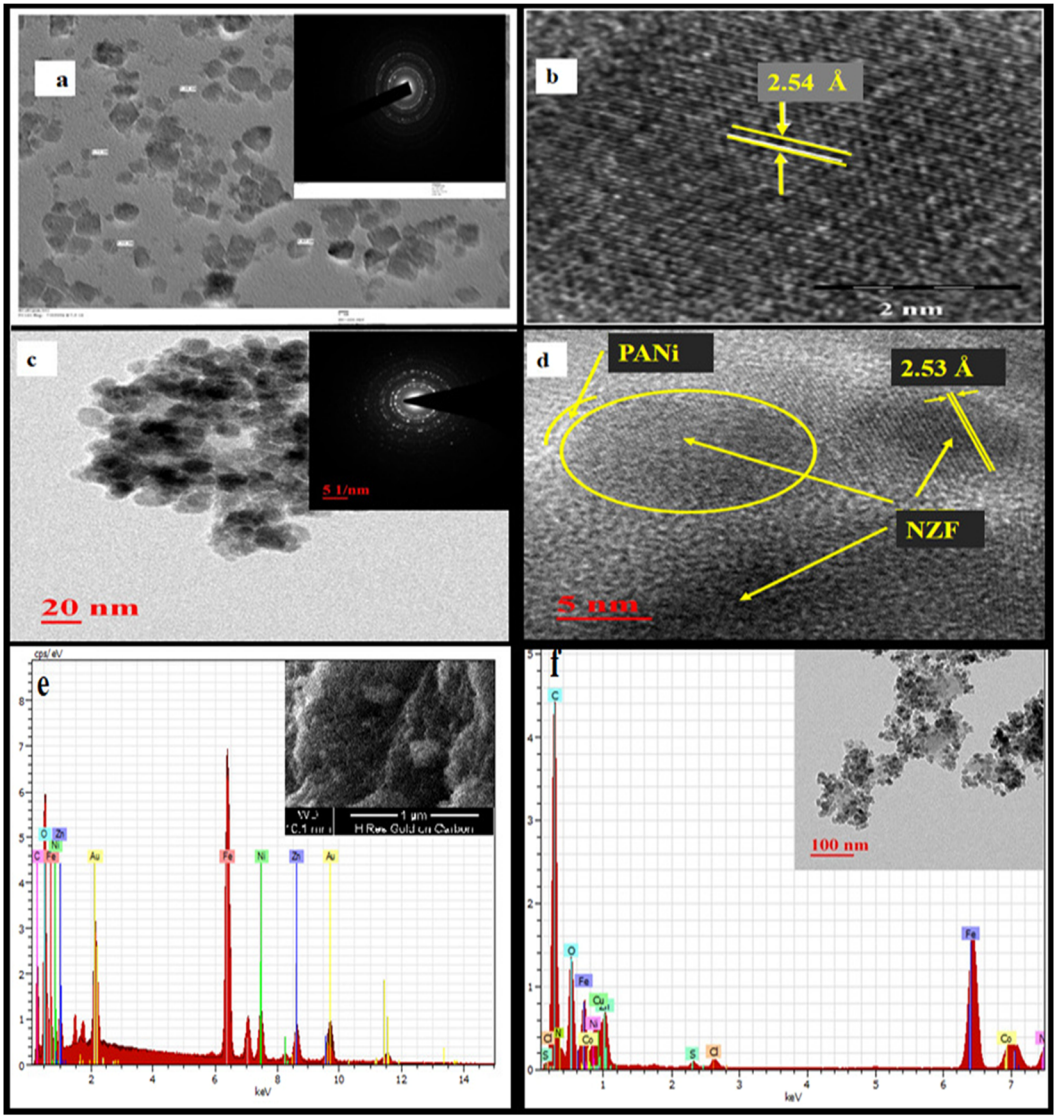
TEM and HRTEM images of NZF (**a**,**b**), NZF@PANi (**c**,**d**) and EDX spectrum of NZF (**e**) and NZF@PANi (**f**).

The Fig. 2(a–f) shows the surface morphologies as well as inner structure of both pure NZF and hybrid NZF@PANi examined by HRTEM. As shown in Fig. 2(a) the pure NZF are almost spherical in shape with average particle size of 11 nm. Whereas the HRTEM image of NZF@PANi, Fig. 2(c), reveals that almost all the particles are successfully enveloped by a thin layer of PANi coating with an average diameter of 14 nm^22^. Further the lattice fringes with a d-spacing of about 2.53 Å can be clearly seen in Fig. 2(d) for the NZF@PANi which is corresponding to the 311 plane of NZF. High resolution TEM images of the hybrid catalyst reveals that the PANi chains are tightly coupled on the surface of NZF nanocrystal by the formation of individual particle shapes and the strong attachment may favour for the charge/electron/hole transfer between NZF and PANi. The selected area electron diffraction patterns (SAED) of both the particles in inset figures demonstrate that the catalysts are crystalline and crystal lattices of NZF particles are intact after *in-situ* polymerization. From the EDX analysis, Fig. 2(e,f), it was confirmed the presence of elements C, O, Fe Ni Zn (base component Au) in NZF pure particles and N, C, O, Fe, NI, Zn (base component Co and Cu) in NZF@PANi hybrids with an approximate Zn:Ni:Fe atomic ratio of 1:1:4. The finding is in fair agreement with the theoretical value for Ni_0.5_Zn_0.5_Fe_2_O_4_.

**Figure 3:**
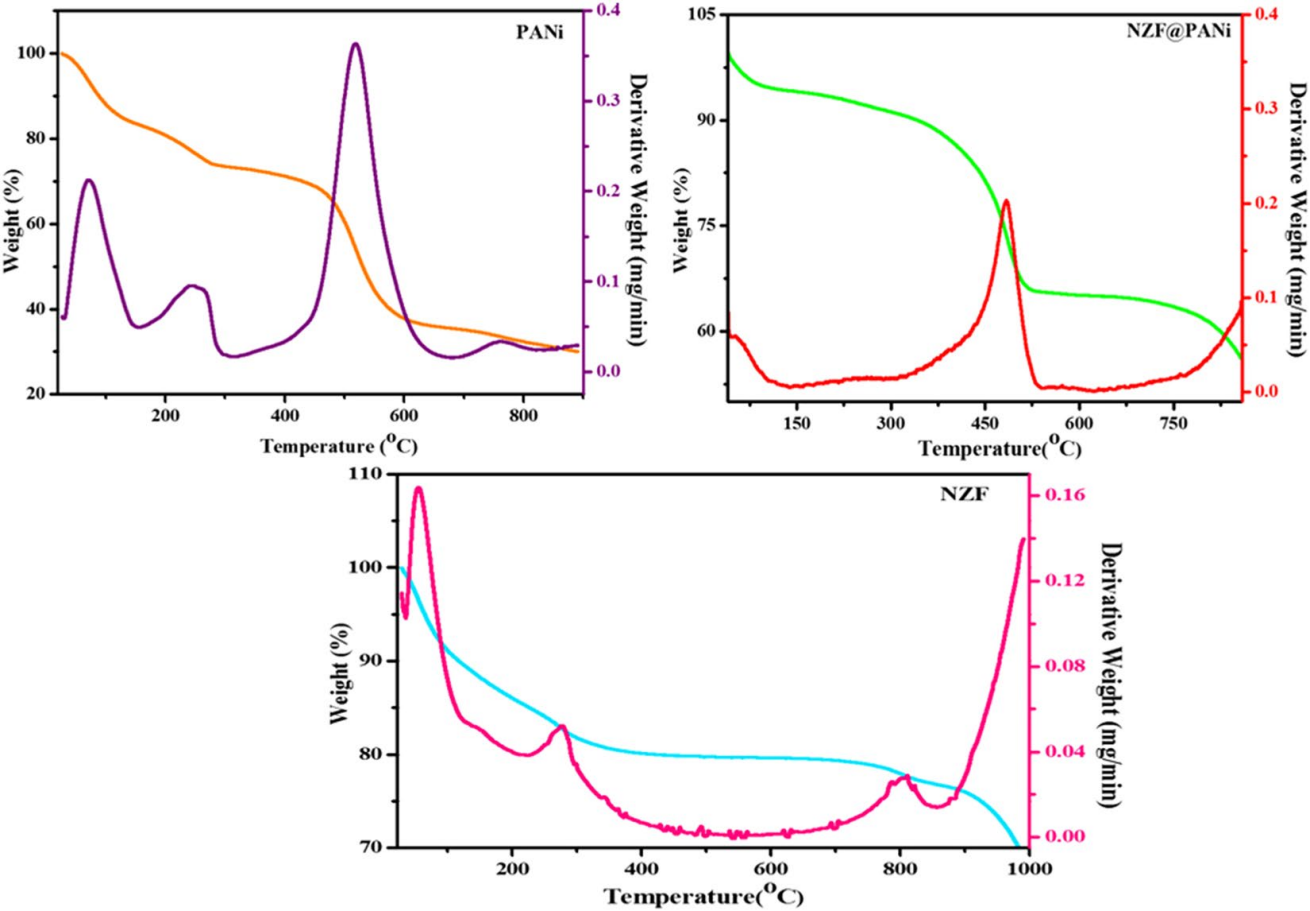
TGA thermograms of pure NZF, PANi and NZF@PANi.

The thermal behaviour of PANi, NZF and NZF@PANi are demonstrated in TGA graphs in the Fig. 3. The three stage degradation of PANi was found in TGA weight loss curves^32^. The first stage weight loss occurred approximately15% at around 70 °C due to elimination of moisture and other low molecular weight volatile substances entrapped in the polymer lattice. In the range of 200 °C to 400 °C, the second stage degradation with weight loss around 10% may be due to thermal decomposition of the low molecular weight oligomers of PANi and HCl^15^. The polymeric nanofibers got finally degraded in the range of 400 °C to 600 °C, contributing the major weight loss of 35%. The TGA result of as prepared NZF conveyed the weight loss of 20% due to evaporation of moisture and surface attached low molecular weight compounds with functional groups of –OH, –COOH compounds as confirmed by FTIR spectra^33^. As for the hybrid NZF@PANi particles, the thermograph discloses the thermal stability and absence of low molecular weight polymer chains of PANi with initial loss around 5% of the total weight corresponding to the moisture and at around 400 °C and above the polymeric nanofibers were degraded contributing approximately 20% of the total weight and remained stable even after 500 °C contributing to rest of the weight percent of the nanocompostie which is analogous to the weight loss observed in TGA of pure NZF particles. Also thermal stability of the NZF@PANi composite portrayed a strong coupling of nanocrystal surfaces and polymer chains^34^.

**Figure 4:**
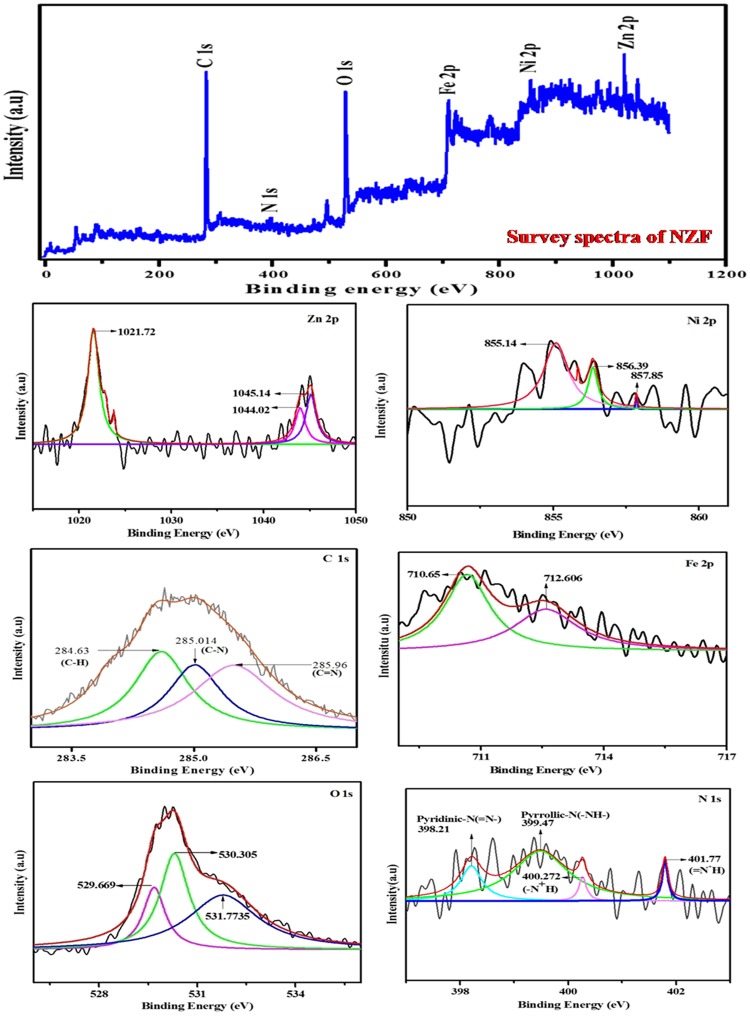
XPS survey spectra of NZF@PANi and XPS pattern of Zn 2p, Ni 2p, C 1 s, Fe 2p, O1s and N 1s.

The XPS survey spectrum, Fig. 4, was conducted to approach the chemical composition and state in NZF@PANi composite. It ensured the presence of C, O, N, Fe and Zn in the as prepared composite particles. The sharp C 1 s peak at 284.63 eV in the composite spectrum (Fig. 4) indicates the presence of non-oxygenated ring carbon of C-N or C=N^35^. In transition metal oxides, the O 1s peak is obtained in the range of 529.669–531.773 eV. In the present survey, the predominant peak of O 1 s at 530.305 eV erected due to metal oxide bond^36^. In Fig. 4, the N 1 s spectrum in the composite can be deconvoluted into four peaks related to different nitrogen forms. The prominent peak at 399.47 eV may be due to some interaction between nitrogen and metal ligand ions^19^ and the peak appearing at 398.21 eV stemmed due to pyridinic-N^37^. The very weak peak at 400.272 eV may be duely assigned to pyrrolic-N and the high binding energy peak at 401.77 eV is associated with the interaction between N^+^ and protons^38,39^. The Nickel 2p_3/2_ photoelectron emission spectra line in the composite with binding energy at 855.14 eV corresponds to that of NiO, indicating the Ni ions are divalent and the Zinc 2p_3/2_ showed their peaks with typical binding energy at 1021.72 eV and 1044.02 eV associated with ZnO^40^. The observations suggest that the valence state of the Ni, Zn substituting the A sites in the regular spinel structure of NZF is not affected by PANi coating. The Fe 2p binding energy peaks were observed at 710.65 eV and 712.606 eV indicating (L3) edge characteristics of the trivalent Fe^3+^ under octahedral crystal field and is as like those of Fe^3+^ oxides indicating most of the Fe ions present in trivalent form^41^. Also, there is an additional peaks of Ni 2p_3/2_ with binding energy of 856.39 eV and Zn 2p_1/2_ corresponding to 1045.14 eV reveals the Ni-N and Zn-N interactions in the composite, without specific interaction with Fe^19^.

**Figure 5:**
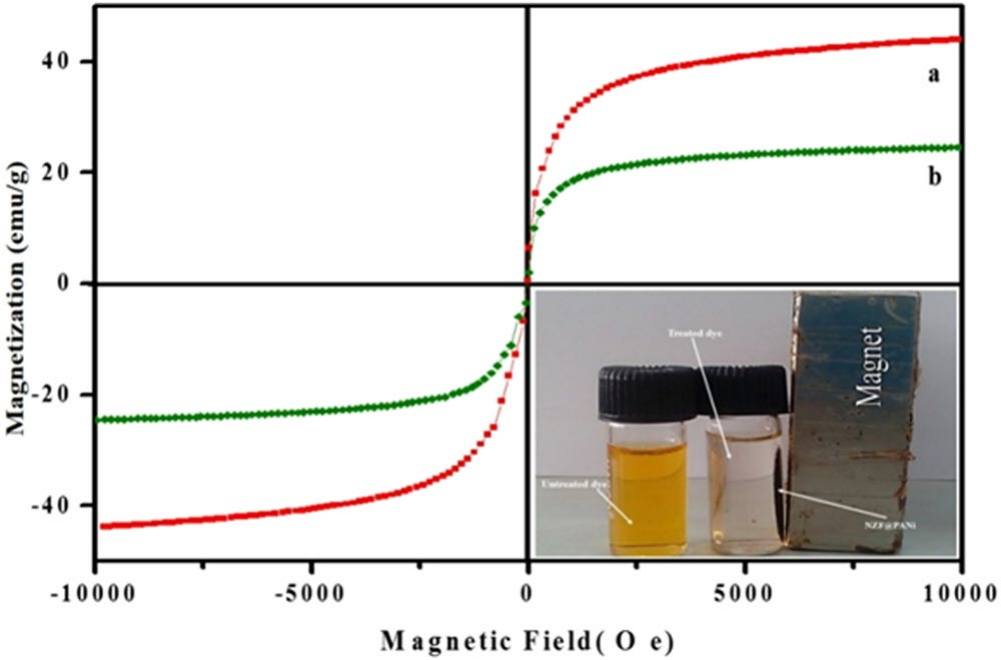
Magnetization curve of NZF (**a**) and NZF@PANi (**b**), the inset shows phototocatalyst separation using an external magnet.

Figure 5 illustrates the magnetic properties of as prepared NZF and the NZF@PANi composites measured at 300 K. Both the materials show typical superparamgnetic character with the saturation magnetization (Ms), remanent magnetization (Mr) and coercivity (Hc) values of NZF and NZF@PANi are 45 emu/g, 8.5 emu/g, 62 Oe and 25 emu/g, 4.5 emu/g, 28 Oe respectively. The saturation magnetization value of the composite is lower than that of NZF due to coating of polymer over it, however the excellent magnetic properties of the NZF was maintained in the composite. Therefore, the composite photocatalyst could be separated easily from the aqueous solution using an external magnetic field as shown in the inset Fig. 5.

**Figure 6:**
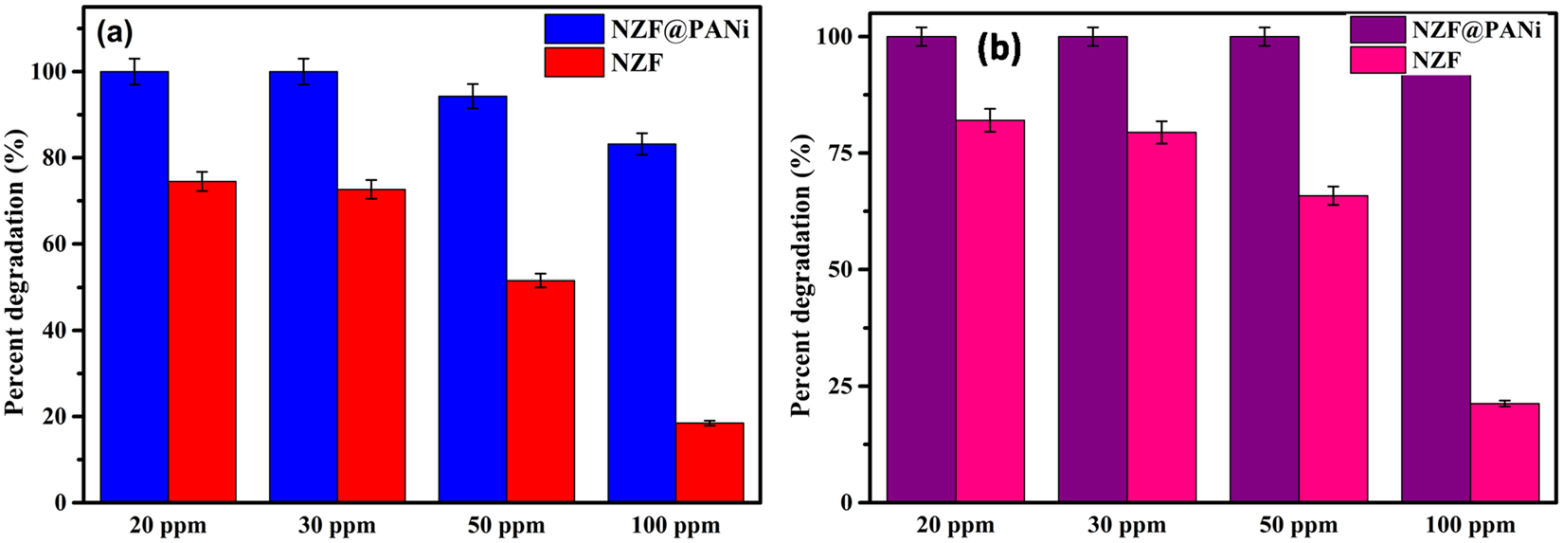
Percent dye degradation at: 5 min (**a**) and 30 min (**b**).

**Figure 7:**
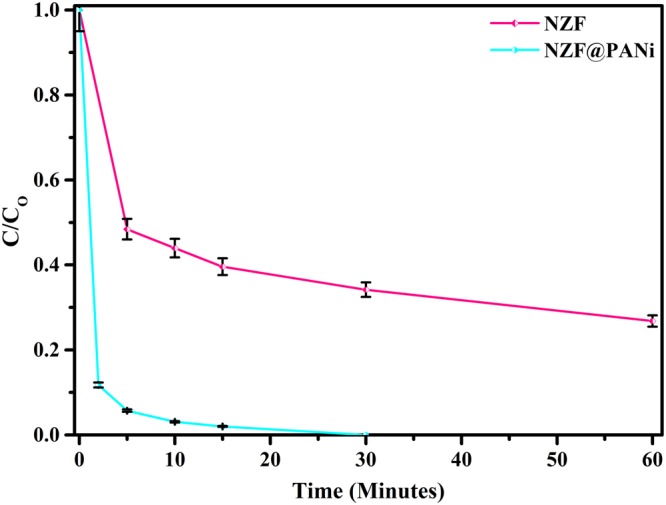
Degradation of 50 ppm orange II under ambient conditions.

The catalytic degradation performance for the liquid-phase aqueous Orange-II, a typical azo dye under ambient conditions without any additional chemical activators or external energy input over NZF@PANi nanocomposites has been demonstrated in Fig. 6. Surprisingly, the developed composite has been found to be a highly efficient catalyst for the degradation of Orange-II under ambient conditions as compared to bare NZF catalyst. In order to demonstrate, the removal of azo dye from waste water is a truly catalytic oxidation or not, the catalyst particles were separated after dye degradation and tested for the leaching of dye molecules in water from used catalyst. It was observed that the leached solution did not impart any colour and manifested any peak on being examined over UV-Visible spectrophotometer. The finding ratifies that the reduction mechanism is to be a catalytic not an adsorption phenomenon. Further, the measured 81% total organic carbon (TOC) removal for the 50 ppm dye degradation for half an hour also supports that the process for the removal of dye to be catalytic in nature, not just a mere de-coloration process^22^. From the Fig. 7 it is observed that the Orange II dye was degraded gradually and scantily in the presence of plain NZF nanoparticles. Only 51.2% and 65.58% degradation of 50 ppm dye was observed at 5 minutes and 30 minutes respectively with plain NZF. However the coating of PANi on NZF not only remarkably boosts the percentage removal but also the rate of removal leaped incredibly to 99% removal in 10 minutes and it degrades almost 100% within 15 minutes for 50 ppm Orange II dye aqueous solution with disappearance of the UV-Visible absorbance peak at 485 nm as shown in supporting information (Fig. S1(a)). The acid Orange II shows the absorbance peak at around 485 nm wavelength. The intensity or the absorbance value of the peak varies proportionally with concentration of the dye present in the solution. As the reaction progresses, the concentration of the dye reduces and hence the absorbance value of the peak decreases. This can be attributed to the degradation of azo bond (-N=N-), which is responsible for the coloration. Besides catalytic activity, the NZF@PANi can also be easily separated using a magnet. On treating 100 ppm dye solution with same amount of catalyst, the reaction rate slowed down, however 97% dye removal was still observed in 30 minutes. The rate of kinetics was determined using 50 ppm dye concentration as shown in Fig. S1(b) and Table S1 (Supporting Information). Also, the degradation of a non-colour toxic organic aquous contaminate, bisphenol-A (BPA) (Fig. S2) has been incorporated to broaden the catalytic activity of the developed catalyst and the TOC removal for Bisphenol A (10 ppm) after 3 hours study at ambient condition and was found 35% to the corresponding BPA removal of 45%. But this could be attributed to its slow kinetics as compared to Orange II degradation. Our result is also comparable with the previous published works which are mostly at accelerated condition either by the presence of light, H_2_O_2_, peroxymonosulphate^42^.

## Discussions

**Figure 8:**
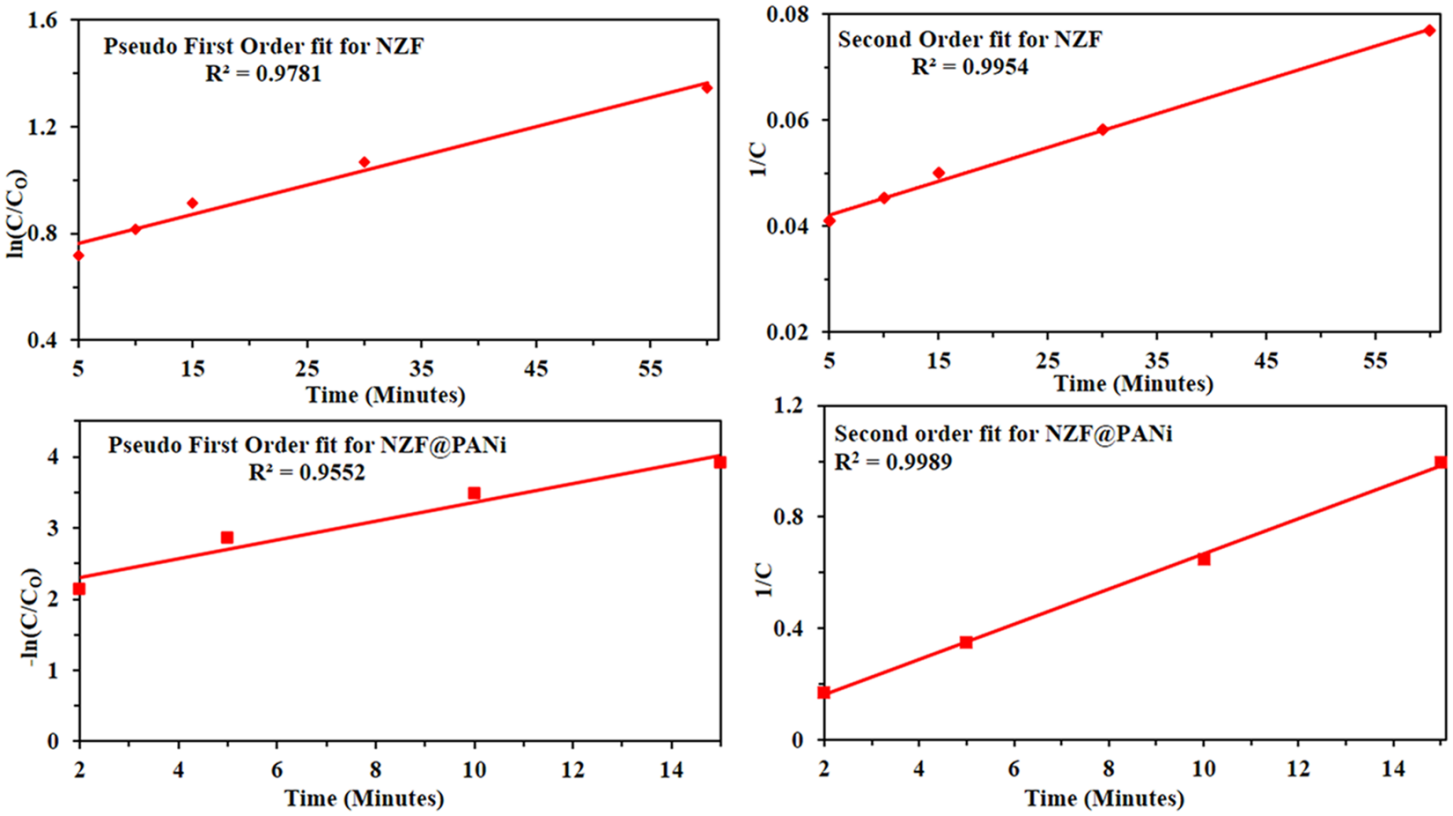
Rate calculation for the 50 ppm dye degradation process with: NZF (**a**,**b**) and NZF@PANi (**c**,**d**).

The synergistic effect of the NZF@PANi composite for the dye degradation dyes in waste water validating the fact that the integration of PANi nanolayer with other semiconductors is a promising technique to boost catalytic efficiency. Many researchers have already reported the synergistic effect of PANi with other semiconductors as e.g. TiO_2_, BiOCl, Ni_2_Fe_2_O_4_, CdO etc., but the present study has found an excellent catalytic performance and is cost effective as compared to the reported results as presented in Table 1. To illustrate the light harvesting capacity of the composite catalyst the aqueous solution of Orange-II was treated in dark, at ambient laboratory conditions and in visible light (500 Watt bulb) and the result is presented in Fig. S3 (Supporting Information). The result explored that the catalyst is sensitive to light sources but at ambient condition the catalyst offer considerable light harvesting capacity in the visible energy region as discernible from the DRS and EIS results presented later on. To the best of our knowledge, there are only a limited works on the photocatalytic degradation at ambient condition with low cost and easy separation of the used catalyst from the effluent. Our studies indicated that the synthesized catalyst is more effective on decolorization of azo dyes under the same condition as reported earlier (Table 1) even at ambient condition. To illustrate the catalytic performance of the bare NZF and NZF@PANi composite more precisely, the kinetics of the degradation with different dye concentrations is presented in Fig. 8. The resultant degradation showed a second order reaction model utilizing the following integral form of the kinetics equation:

2 $$\frac{1}{C}=kt+\frac{1}{{C}_{o}}$$

where C_0_ and C are the dye concentration of Orange-II at initial time and at time interval t under ambient condition and k is the degradation constant of the second order reaction. The analysis of the degradation constant signify that the composite catalyst offer the best degradation constant 3.864 (l/mg)^−1^ h^−1^. The commendable catalytic performance of the developed catalyst could be credited to the integrating synergistic performance with Type-II band structure and made the catalyst for enhanced light harvesting ability. To manifest the pH effect of waste water, the catalyst was exposed to the different pH and the result is shown in Fig. S4 (Supporting Information). The result demonstrate that the dye degradation is almost unaffected by the solution pH and could be used in a wide range of pH.

**Table 1:** Comparison of catalytic performance of NZF@PANi composite with other reported polyaniline (PANi) coated nanocomposites.

Composite	Irradiat-ion source	Dye	Degra-dation time.	Dye concentrate-ion	Catal-yst dose	% Degrada-tion	Reference, year of publication
Ni_0.5_Zn_0.5_Fe_2_O_4_@PANi	Ambient condition	Orange-II	15 min, 60 min	50 mg/l, 100 mg/l	1 g/l, 1 g/l	100 98.1	This study
PANi modified TiO_2_	Xenon, 500 W	MO	360 min.	10 mg/l	1 g/l	96	^17^, 2012
Titania-CoFe_2_O_4_-PANi	Visible light	MO	420 min	40 mg/l	0.25 g/l	70	^59^, 2013
Polyanililine/CoFe_2_O_4_	UV	MO	240 min	10 mg/l	0.05 g/l	90	^60^, 2013
Polyaniline/CdO	Natural Sunlight	MB, MG	240 min	1.5 × 10^−5^ M	0.4 g/l	99	^50^, 2013
Polyaniline- Hybrid Defective ZnO	UV	MO	120 min	3 × 10^−5^ M	0.5 g/l	97	^51^, 2014
PANI-modified BiOCl	Xenon, 500 W	MO	210 min	10 mg/l	1 g/l	67	^61^, 2013
Cobalt ferrite-polyanilinenanofiber	UV	MO	120 min	20 mg/l	0.2 g/l	85	^21^, 2016
Cobalt ferrite-polyaniline	Xenon, 500 W	MO	480 min	20 mg/l	0.25 g/l	80	^19^, 2012

**Figure 9:**
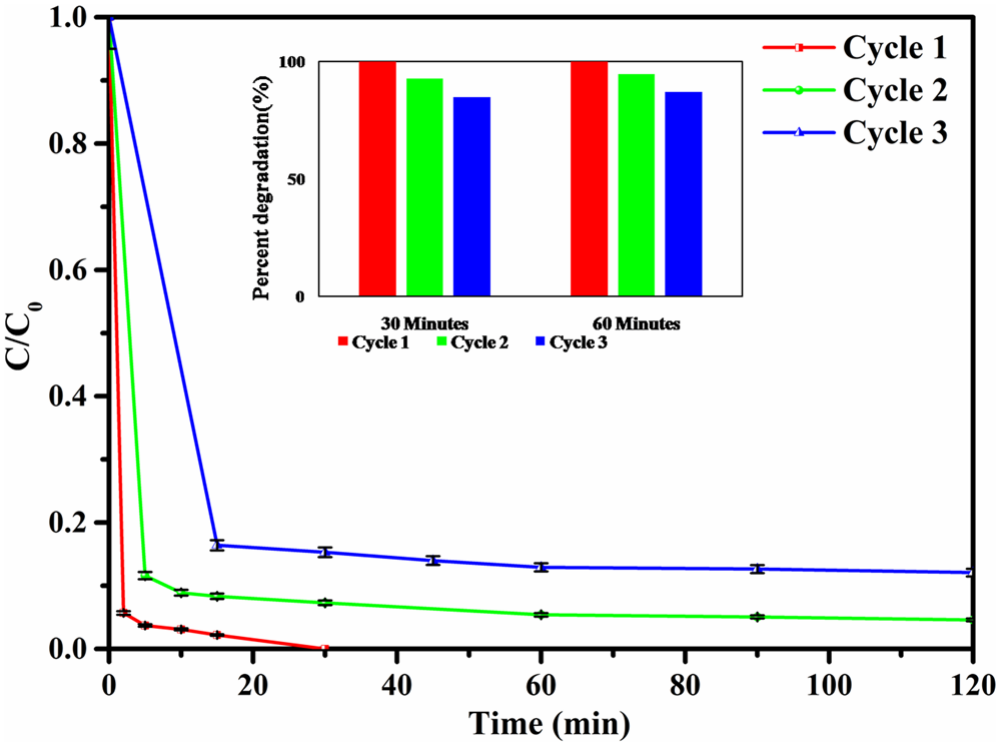
Recycle study of NZF@PANi in the form of degradation kinetics for 50 ppm dye solution.

One of the cardinal parameter in photocatalysis process is the catalytic reusability, as it affects the cost of treatment process significantly^43^. As shown in Fig. 9, our catalyst activity was studied for three cycles of experiments. In the first run the dye degraded almost completely within 15 minutes and showed 100% degradation in 30 minutes, 94.6% efficiency for second run and 88% for the third run. The downtrend of photo catalytic degradation rate could be a result of blockage of active sites due to intense adsorption and catalytic degradation on the heterogenous catalyst surfaces^17^. Moreover, the physiochemical stability of the recycled catalyst was further inspected by XRD and SEM (Fig. S5). XRD results confirm that phase and structure of the catalyst remained unchanged. Also, SEM image of the recovered photocatalyst were barely changed.

It is very interesting that NZF alone was catalytically very weak to degrade azo dye in aqueous phase, whereas the integration of PANi and NZF leads to a dramatic enhancement in the catalytic degradation under ambient conditions. This exceptional and significant enhancement in catalytic dye degradation activity can be attributed by the remarkable synergistic effect of NZF and PANi due to transformation into a single integral nanostructure catalyst. It is well versed and accepted that the adsorption capacity is an inherent factor of the photocatalyst to influence the catalytic activity^44,45^. It is also well reported that the photocatalytic behaviour of the pure PANi under natural light is a very weak catalyst and this can be accredited to the photoexcitation of quinoid segments of the PANi emeraldine form through π-π* transitrion^46,47^. But the PANi based heterostructured photocatalyst having positively charged backbone of PANi adsorb anionic dyes like Orange II more effectively due to charged-charged interaction and enhances the rate of degradation^26^.

**Figure 10:**
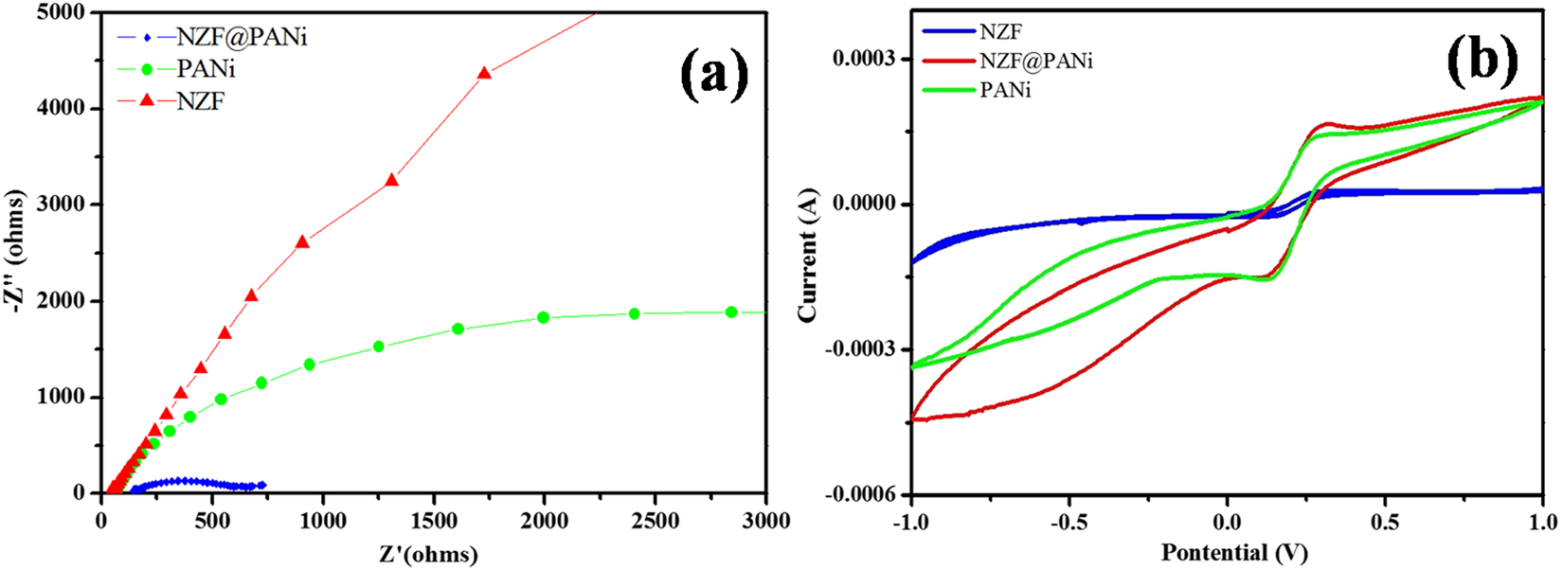
EIS Nyquist plot (**a**) and Cyclic Voltametric test (**b**) for NZF, PANi and NZF@PANi.

Furthermore the increase in photocatalytic activity in PANi based heterostructured nanoscale catalysts may be due to the exceptional light harvesting photosensitivity and generation of electron hole pairs^48^. Therefore to understand the synergistic effect for the significant enhancement in the catalytic capacity of NZF@PANi composite it is tried to figure out the separating and transferring efficiency of the charge carriers through the electrochemical impedance spectroscopy (EIS) measurements. The EIS is a fundamental technique to characterise the interfacial charge transfer properties of a material and it is accepted that the diameters of semicircles in Nyquist plots are equal to charge transfer resistance of the electrode surface and a smaller arc radius means more effective separation of the photogenerated electron-hole pairs and a faster interfacial charge transfer^49,50^. Figure 10(a) displayed the Nyquist plot for the as prepared NZF, PANi and NZF@PANi composites. The composite showed a very smaller arc radius as compared to pure NZF and PANi and it was consistent with the result of PL analysis. Findings indicate that the composite NZF@PANi can separate and transfer charge carriers more effectively than pure NZF, thus leads to the significant photocatalytic performance^50^. The cyclic voltammeter (CV) responses of NZF, PANi and their composite were recorded in 5 mM of [Fe (CN)_6_]^3−/4−^ in 0.1 M KCl at a scan rate of 5 mV/s as shown in Fig. 10(b). The photocurrent intensity versus potential of NZF@PANi confirmed that the photocurrent density at ambient condition in positive bias potential was much higher than that of NZF. The cyclic voltmeter study and EIS measurement at ambient laboratory light indicates that the measured current of the NZF@PANi (0.225 mA) is much higher than that of pure NZF (0.055 mA). It again concludes that the composite catalyst provides appropriate electronic channel and enhances separation of photoelectrons and holes as already supported by EIS and PL studies. Very recently, it is reported that the presence of PANi in the heterostructured composite electrodes of magnetic materials had good electrochemical performance due to its unique hierarchical structure, a short pathway for ion penetration and a connection to the PANi chains and multiferrite oxides^19,51^.

**Figure 11:**
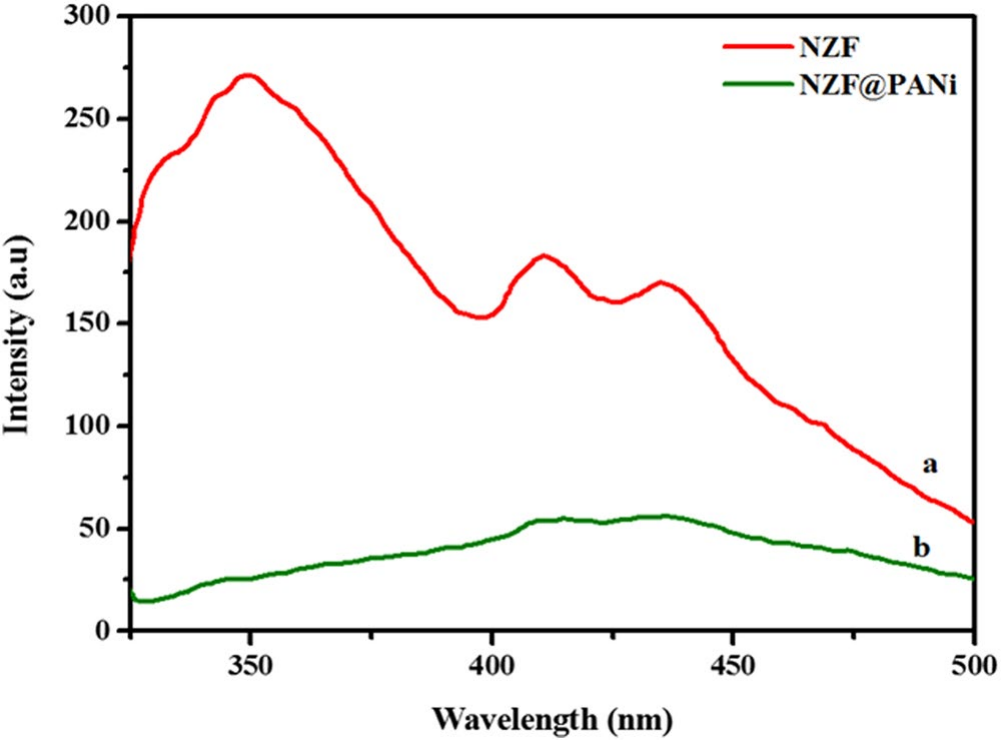
Photoluminescence spectra of the NZF(**a**) and NZF@PANi (**b**).

The photoluminescence spectral (PL) analysis is also commonly used to configure the rate of recombination, life time and the nature of photogenerated electron-hole pairs in the photocatalysts. Here to check the role of the integrated PANi and NZF at room temperature and ambient condition, PL spectra were studied and as presented in Fig. 11. A PL spectrum of bare NZF was attributed to three variable emission spectra within 300 nm to 500 nm. A broad band emission at 350 nm in the UV-region was associated with the intrinsic physical origins of self trapped crystal lattice excitation. The peak at 412 nm was the blue violet regions which originates from surface state defects and assigned to the charge transfer between Fe 3p, Ni 2p and Zn 2p at octahedral sites and its surrounding oxygen ions^52^. The PL emission spectral of NZF showed another excitation band at 428 nm in the blue area that may be attributed to the recombination of holes and electrons in the valence and conduction band due to different intrinsic defects such as vacancies, metal vacancies, mean interstitial defects^53^. However after PANi coating on NZF, the PL emission intensity band peaks in the whole region was almost vanish as shown in the Fig. 11. The very sharp quenching of the fluorescence spectra is supposed to stem from the separation of charge carrier on the integral interface between PANi and NZF. This strong emission quenching is also positively hypothesised that the integration of PANi and NZF strongly influence the charge transfer process^54^.

A major factor for accounting the origin of commendable catalytic efficiency in NZF@PANi composite may also be due to the core-shell interfacial band structure and that may further provide an additional degree of freedom for the enhancement in spectral response^55^. It is observed that the core-shell nanostructure interface shows an indirect transition at higher wavelengths that cannot be provided by individual components. Additionally this may also promote the suppression of the electron (e^−^) – hole (h^+^) recombination. The elemental mapping result is reported in Fig. S6 (Supporting Information) and it confirmed the core-shell structure of NZF@PANi heterostructure catalyst. Further, the UV-vis-DRS spectra (Fig. S7, Supporting Information) of pure NZF, PANi homopolymer and NZF@PANi composite were performed to execute the optical properties and light harvesting ability. The adsorption spectra of PANi and NZF@PANi divulge the similar characteristics bands as usual three adsorption bands at 320–350, 400–450 and 650–700 nm corresponding to the π-π* electron transition in the benzenoid segments and the formation of polarons in quinoid segments^21^. Although the coating of PANi on NZF as shell side, there is red shift to higher wave length in the corresponding spectrum for the composite. The calculated optical band gap (Eg) values were 1.85 eV, 1.42 eV and 1.63 eV corresponding to NZF, PANi and NZF@PANi. The shifting of onset adsorption of NZF to longer wave length and wide spread broadening of the absorption edge due to PANi coating on the NZF may be due to the core-shell geometry with improved interfacial interaction and confine the photoinduced charge carriers in the core as well as in shell, leading to suppression in the recombination rate^56^. Thus, the DRS results discern that the developed composite achieved considerable light harvesting capacity even at ambient conditions due to interfacial integration between NZF and PANi as compare to their individual role.

**Figure 12:**
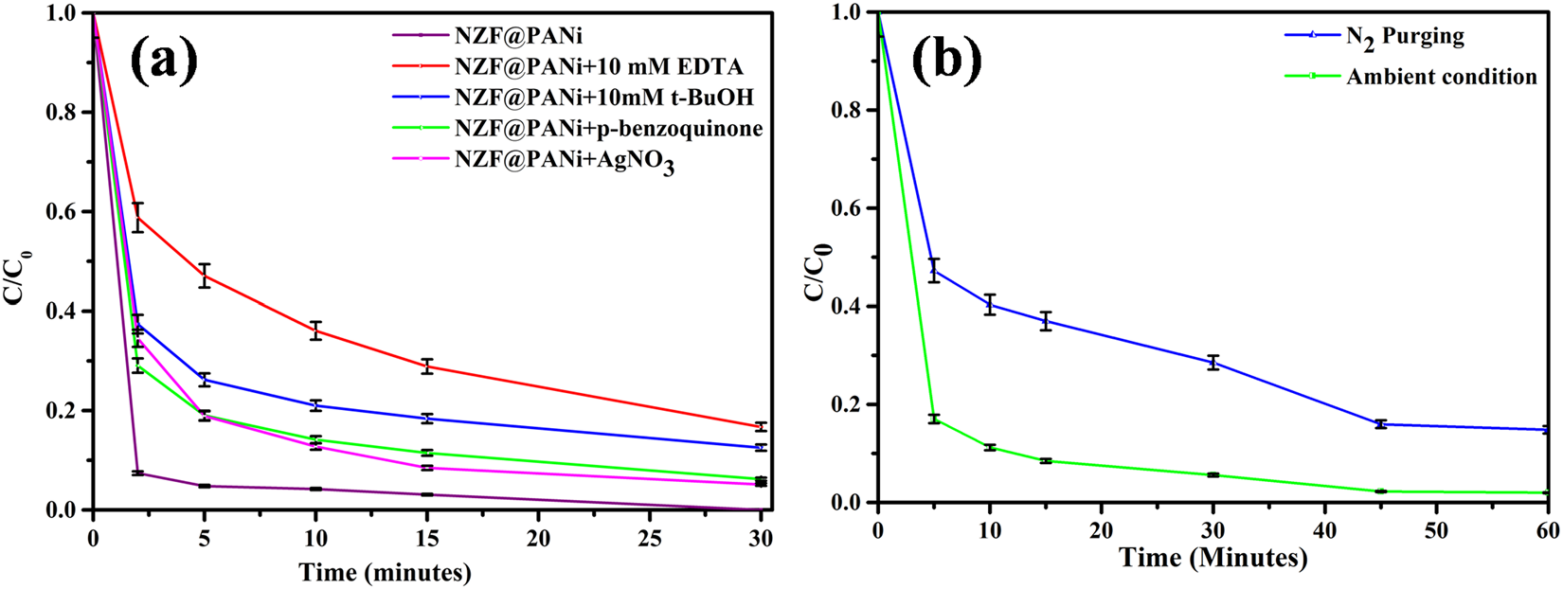
Effect of different scavengers (**a**) and dissolved oxygen (**b**) on catalytic activity of NZF@PANi at ambient conditions.

It is emphasized that the separated charge carriers i.e., the excited electrons from the valence band (VB) to the conduction band (CB) and generated holes in the VB are the origin for photocatalytic degradation by the semiconductors through underlying redox reaction on the catalyst surface^57^. These generated electron-hole pairs (e_CB_^−^/h_VB_^+^) on the catalyst surface react with the adsorbed species and generate active radicals such as hydroxyl radicals (OH.) and superoxide radicals ions (O_2_^−^) through redox reaction. These radicals participate in the oxidative degradation of the absorbed dye molecules on the catalyst surface. Figure 12(a) shows different experimental kinetics in presence of specific reactive species scavenging chemicals. The figure clearly demonstrates that the degradation rate decreased extensively on addition of EDTA-2Na and t-ButOH, suggesting the active role of the holes and the generated of hydroxyl radical (^.^OH) significantly participate in the dye degradation. The degradation rate moderately lowered on addition of benzoquinone signal that superoxide radicals were responsible for degradation to some extent while addition of AgNO_3_ had little or negligible effect on degradation rate proving electrons were meagrely produced and very weakly influenced the rate.

Another way, it is hypothesized that the reduction of dye-stuffs using semiconductor catalysts may utilise dissolve oxygen that generates superoxide radicals (O_2_^.−^) or reactive oxygenated species, which further convert rapidly to H_2_O_2_. The generated H_2_O_2_ produces secondary radicals OH^.^ thereby causing oxidative cleavage of dye molecules and generate hydroxylated products^58^. Recently, it is manifested that the generation of reactive oxygenated species combined with dissolved oxygen in photochemical process indeed plays a key role for the oxidative cleavage of organic pollutants. Figure 12(b) shows the degradation of Orange II dye by the developed catalyst in presence (in common double distilled water) and absence of oxygen (purged with N_2_ for half an hour). Preliminary results reveal that the removal of Orange II dye from its aqueous solution is indeed dependent on the generation of reactive oxygenated species and degradation is an oxidative process. It is well implicit that the hydroxyl radicals are formed with the absorbed oxygen on the solid catalyst surface (support by XPS result) that carry out oxidation of dye molecules present in aqueous phase and this is also supported by the scavenger experimental results.

Based on the above studies the following reaction pathways are proposed. The reaction equation (4) predict the generation of electron-hole pairs and the reactions pathways from equations (5) to (6) explore the formation of active radicals that prevent the recombination of e_CB_^−^/h_VB_^+^ and increase the catalytic activity of the catalyst. The produced active radicals can then react with the dye molecules to form the degradation products as predicted in the equations (7), (8) and (9). Another mechanistic reaction pathway has also been comprehended by many researchers that the presence of adsorbed dye molecules on the catalyst surface prolonged in the effluent under natural light may sensitize photocatalytic process through excitation of dye molecules and consequent electron transfer to the conduction band of the photocatalysts^26^.

3 $$PANi+ambient\,light\to PANi(h+{\rm{e}})$$

4 $${\rm{PANi}}\,({\rm{e}})+{NZF}\to {PANi}+{\rm{NZF}}\,({\rm{e}})$$

5 $$NZF\,({\rm{e}})+{{\rm{O}}}_{2}\to {}^{\cdot }O_{2}+NZF\,(h)$$

6 $$NZF\,(h)+PANi\to PANi\,(h)$$

7 $$PANi\,(h)+O{H}^{-}\to PANi+{}^{\cdot }OH$$

8 $$PANi(h)+{}^{\cdot }{\rm{O}}{\rm{H}}+{}^{\cdot }{\rm{O}}_{2}\to Degraded\,products$$

**Figure 13:**
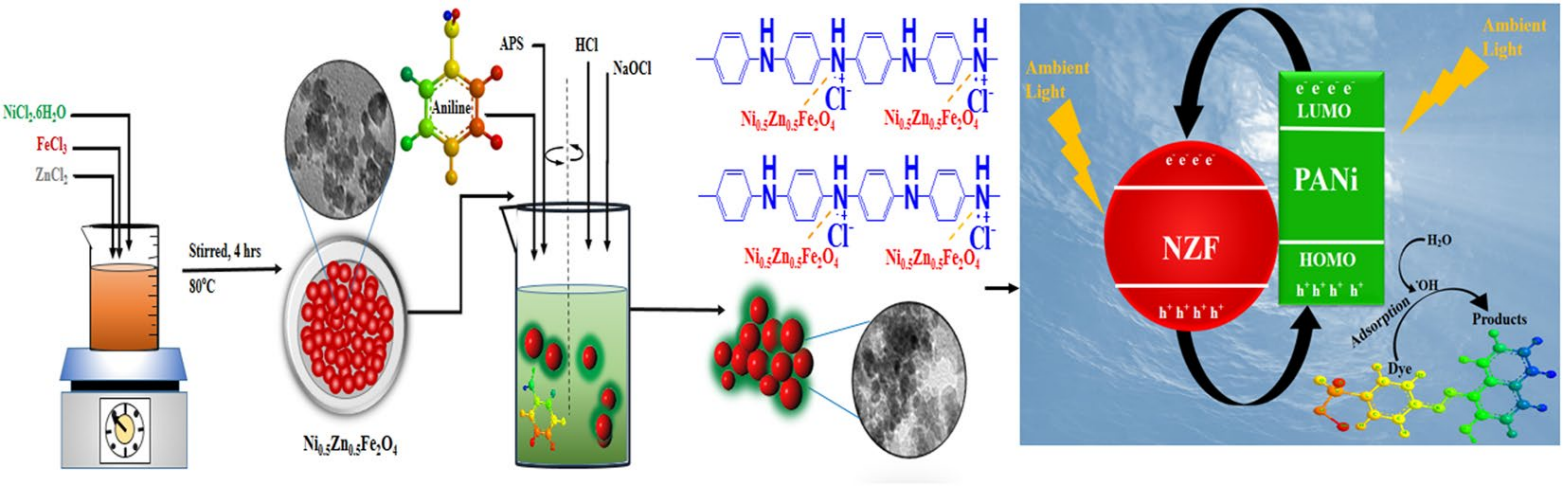
Schematic diagram representing mechanism of dye degradation.

The protagonist of the fast kinetics of dye reduction is the synergistic interaction between PANi and semiconductors^59–61^. PANi being an excellent electron donor and proficient holes carrier enhances the dye reduction capability of plain NZF particle to a very large extent^19^. The available light under ambient conditions caused the excitation of electrons from HOMO to LUMO of PANi matrix and the generation of charge carriers in NZF particles with the electrons jumping to the conduction band and simultaneously generated holes in valence band band that migrate to the backbone of PANi. The PANi, being an excellent electron donor transfers its LUMO electrons to the conduction band of NZF particles, and also readily accepts the holes from NZF as a proficient holes carrier, leading to plenty of charge availability in its positive backbone of PANi (the zeta potential value of the heterostructured photocatalysts was found +30.1 mV and confirmed the positive charges on the surface) and conduction band of NZF. The positively charged PANi backbone causes the adsorption of dye on its surface, which is then degraded by the hydroxyl radicals generated when water molecule reacts with a hole of PANi. Based on the above information, findings and discussions, we have comprehended a probable schematic interpretation for the formation of integrated heterostructured nanoscale NZF@PANi catalyst and for the interaction accounting for an enhanced catalytic activity at ambient conditions as shown in Fig. 13.

## Conclusions

Collectively the present investigation corroborates that NZF@PANi, an integrated heterostructured nanoscale photocatalyst, has been successfully developed for potential degradation of Orange-II dye with utilization of ambient light absorption and retardation of induced charge carriers in the gradient band structure as well as with easy separation. Summarized results acquired from the PXRD, FTIR, SEM, TEM, HRTEM, XPS, EDX, DRS and EIS studies conclude the realization of successful integration of composite nanostructured NZF@PANi photocatalyst and synergistic role for benchmark catalytic degradation of organic dye. It is plausible that the PANi accelerates the adsorption of anionic dye as well as increase the light capturing efficiency. The propitious catalytic performance at ambient conditions could be credited to interfacial interaction between PANi chains and heterometal ions and may ensures the easy light harvesting, accelerate transportation and reduction in recombination of electron and holes as evidence from photoluminescence and EIS. Also insight into the synergistic role for the dye degradation has been discerned through parametric and trapping experiments studies and based on our findings a credible degradation mechanism has been proposed. Thus, it can be argued that the developed catalyst presents a promising material into the photocatalytic domain for addressing the environmental depollution of aquatic dye pollutants.

## Methods

### Chemicals

All the chemicals were of analytical reagent grade. Ferric Chloride (FeCl_3_.6H_2_O), Nickel chloride (NiCl_2_.6H_2_O), Zinc chloride (ZnCl_2_) and Ammonium persulfate (APS) of Merck Germany were used. Sodium Hydroxide Pellets (NaOH), acetone and hydrochloric acid were procured from Fisher Scientific. Aniline, Sodium Hypochlorite and Orange II sodium salt dye were used taken from Sigma Aldrich. The double distillate water was used for experimentation.

### Synthesis of Magnetic Ni_0.5_Zn_0.5_Fe_2_O_4_ Nanoparticles

Nickel Zinc Ferrite (NZF) nanoparticles were prepared by mixing each 100 ml aqueous solution of 1 M FeCl_3_, 0.25 M NiCl_2,_ 6H_2_O and 0.25 M ZnCl_2_ and 250 ml of 2 N NaOH in a beaker placed over magnetic stirrer at 1500 rpm and boiled at 80 °C^23^. The pH was maintained around 12 by adding NaOH solution and kept for 4 hours. The synthesized nanocrystals allowed to be settled after cooling using a strong magnet and supernatant was decanted. The precipitate obtained was washed repeatedly till the supernatant became colourless. The washed precipitate was dried in an oven at 80 °C overnight for further usage.

### Synthesis of PANi coated nanoparticles (NZF@PANi)

The NZF@PANi hybrid nanoparticles were synthesized by the chemical oxidation *in-situ* polymerization of aniline in the presence of dispersed Ni_0.5_Zn_0.5_Fe_2_O_4_ colloidal particles at 4 °C kept in an incubator shaker, shaking at 200 rpm. APS was used as oxidant^15^. In a typical procedure, 0.15 g of as prepared NZF nanoparticles were taken in 6.7 ml of 1 N HCl in four 30 ml glass vial, sonicated for 20 minutes to reduce the aggregation of the magnetic particles. In this solution, 0.15 ml of aniline and 6.7 ml of 0.079 M solution of APS were added. After 6 minutes of addition of the aniline, 0.1 ml of NaOCl (5% by weight) solution was added in the same mixture. The solution was then left in a shaker without disturbing them for 1 hour at the same temperature. The dark green precipitate of prepared hybrid composite were separated by using a strong magnet and washed repeated with water and acetone. Finally, the dark green fine powder of the catalyst was obtained by drying at 40 °C for six hour in a vacuum oven.

### Characterization

The crystalline patterns of the composites were obtained by X-Ray diffraction (XRD) powder analyser on a Rigaku Ultima IV, equipped with Cu Kα radiation having a wavelength of 1.54 Å. The sample was scanned through a 2θ range of 10–90° at a rate of 8°/min. The chemical nature of the NZF and NZF@PANi surfaces were analysed by Fourier Transform Infrared (FTIR) spectrophotometer-3000 Hyperion Microscope Vertex 80. The FTIR spectra were obtained from KBr pressed pellets in the range 4000-400 cm^−1^ with 1 cm^−1^ resolution. The surface morphology and chemical composition of as-prepared nanoparticles and composites were carried by a Zeiss model EVO40 scanning electron microscope equipped with an energy dispersive X-ray (EDX) spectrophotometer. The particle size and inner structure was recorded by a high resolution JEOL JEM 2100 F transmission electron microscope. The chemical state of the as prepared catalyst was performed by X-ray photoelectron spectroscopy (Escalab 210 system, Germany) with a monochromatic Al Kα radiation source. The thermal properties were determined by a Q500 V20.10 Build 36 differential thermal analyzer (TG-DTA) in nitrogen atmosphere at heating rate of 10 °C min^−1^ from room temperature to 900 °C. The optical band gap energies of the powders were measured by UV-Vis diffuse-reflectance spectra in wavelength range from 200 nm to 800 nm, obtained by Varian Cary 5000 UV-visible spectrophotometer. The photoluminescence (PL) spectra of the as-prepared composites were measured using a fluorescence spectrophotometer (F-4600, Hitachi). To measure photocatalytic activity and optical absorbance of dye solutions, Hitachi U-2900UV-vis spectrophotometer was used. EIS analysis was performed on a Galvanostat potentiostat (SI 6143 instruments).

### Dye reduction procedure

The catalytic activity of NZF@PANi was assessed for degradation of different doses of orange II sodium salt azo dye. The aqueous solutions of 30 ppm, 50 ppm and 100 ppm dye were the chosen dye dosages for the study. In each run, 0.1 g of catalyst was added to 100 ml of dye solution. The nanocatalysts were constantly contacted with dye solution under 200 rpm using orbital shaker. At given time intervals, the sampling of suspension was done and particles were separated out with help of a strong magnet, by placing the samples over it. The supernatant was then studied over UV-Visible spectrophotometer for calculating reduced concentration of the dye. The pH effect was also examined for 100 ppm dye degradation. The catalytic reduction rate was calculated using following equation:

9 $${\rm{D}}( \% )=\,\frac{{\rm{Co}}-{\rm{C}}}{{\rm{Co}}}\,\times 100\,$$

where, D is % dye degradation, Co is initial concentration of dye and C is the final concentration of dye.

The hybrid nanocatalyst was checked for its recyclability by repeated application of the used catalyst. The experiments were performed similar way as mentioned above. After each cycle, the nanocatalyst was washed 3–4 times thoroughly with distilled water to eliminate residual dye and separated using a strong magnet and vacuum dried at 40 °C for 6 hours. The reactive species such as hydroxyl radicals (˙OH), electron (e^−^), holes (h^+^) and superoxide radicals (˙O_2_) generated during catalytic degradation process were determined by adding various scavengers. t-Butyl alcohol (10 mM), disodium ethylenediamine tetraacetate (10 mM), benzoquinone (10 mM) and silver nitrate (10 mM) were chosen as hydroxyl radical (˙OH) scavenger, holes (h^+^) scavenger, superoxide radical (˙O_2_^−^) scavenger and electrons (e^−^) trapper respectively. The trapping experiments were experimented as reported earlier during the photocatalytic activity test^62^.

## Electronic supplementary material

Supplementary Information
