# Supplementary material for: A magnetically recyclable photocatalyst with commendable dye degradation activity at ambient conditions
Source: Sci Rep. 2018 Oct 2;8:14700. doi: 10.1038/s41598-018-32911-3 (PMC6168602; doi:10.1038/s41598-018-32911-3)
Supplement: Supplementary file 1 — Supplementary Information [file 41598_2018_32911_MOESM1_ESM.docx]

**A magnetically recyclable photocatalyst with commendable dye degradation activity at ambient conditions**

**Abhilasha Pant^1^, Ruchika Tanwar^1^, Bikramjit Kaur^1^ and Uttam Kumar Mandal^1^**

^1^University School of Chemical Technology, G.G.S. Indraprastha University, Sector 16C, Dwarka, New Delhi-110078, India

**
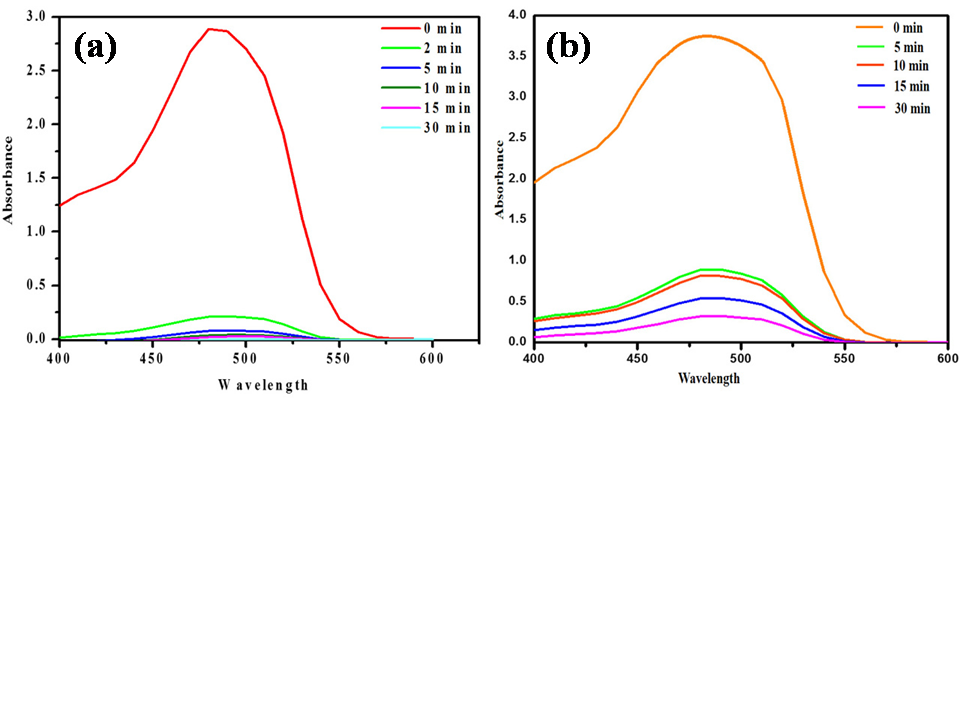
**

**Figure S1.** 50 ppm dye reduction kinetics (a) and 100 ppm dye reduction kinetics (b) using UV-Visible spectrophotometer.

**

**

**Figure S2.** Degradation of 10 ppm BPA using NZF@PANi composite.

**

Figure S3.** Study of light effect using 100 ppm dye solution for NZF@PANi composite.

**
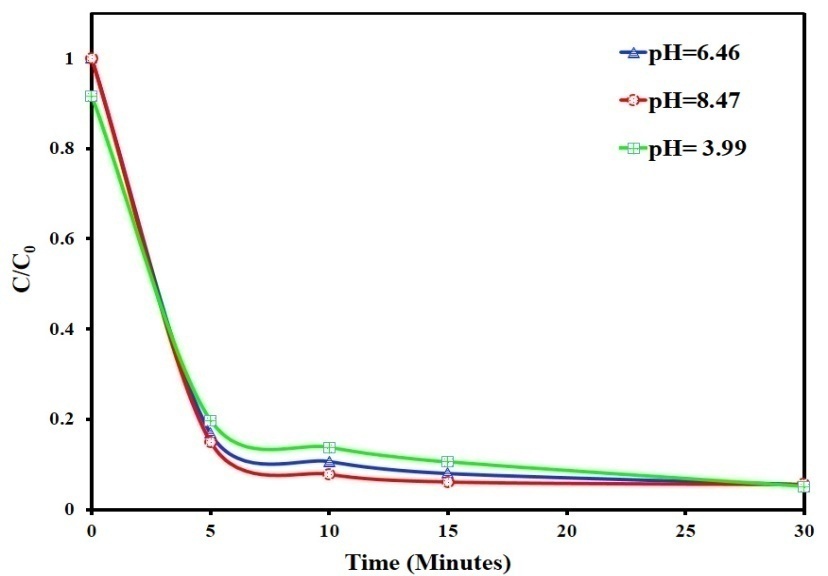
**

**Figure S4.** Effect of pH on degradation of 100 ppm dye in presence of NZF@PANi under ambient environment.


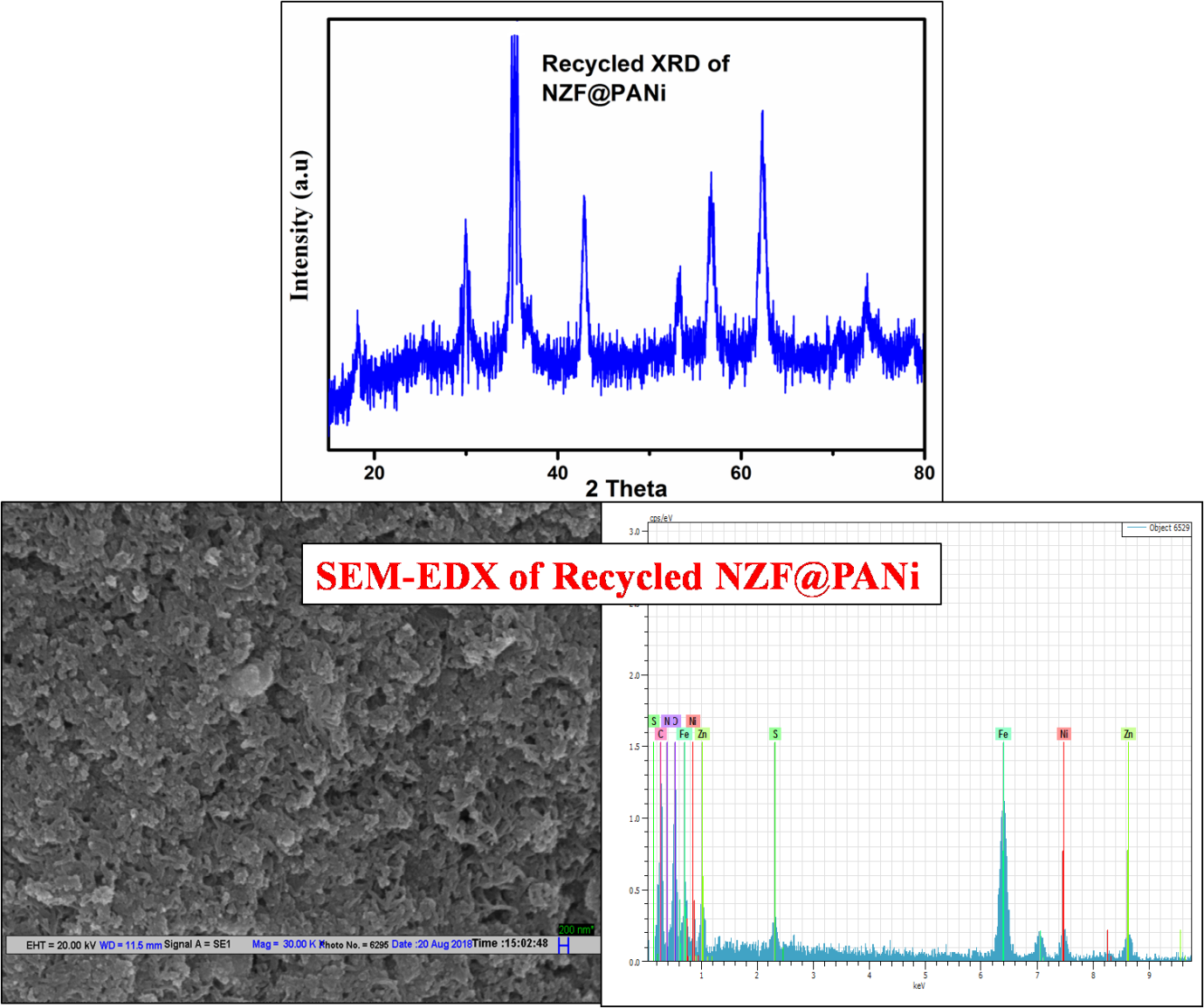


**Figure S5.** XRD and SEM-EDX spectra of recycled NZF@PANi


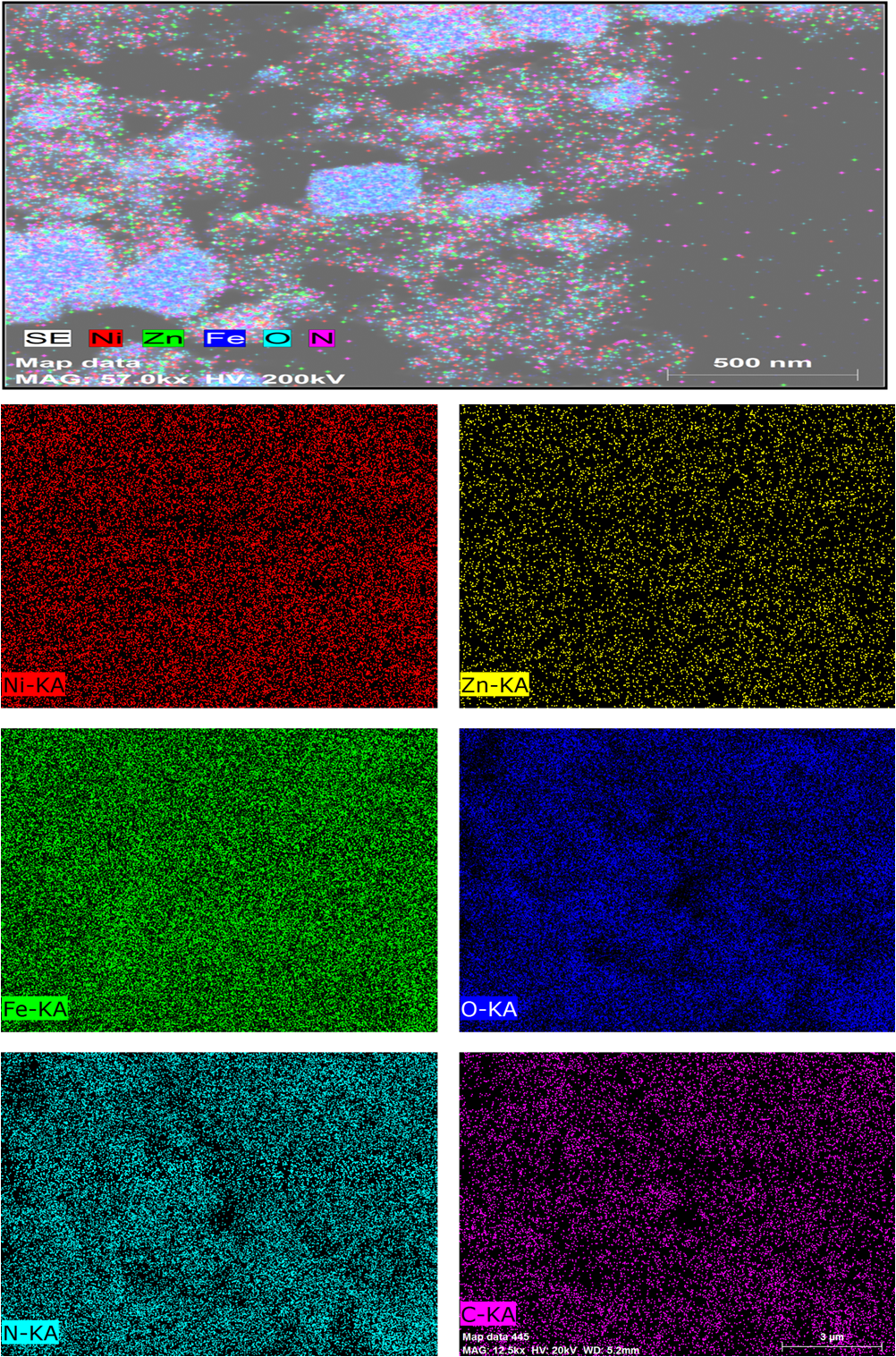


**Figure S6.** Elemental mapping of the NZF@PANi composite.

**
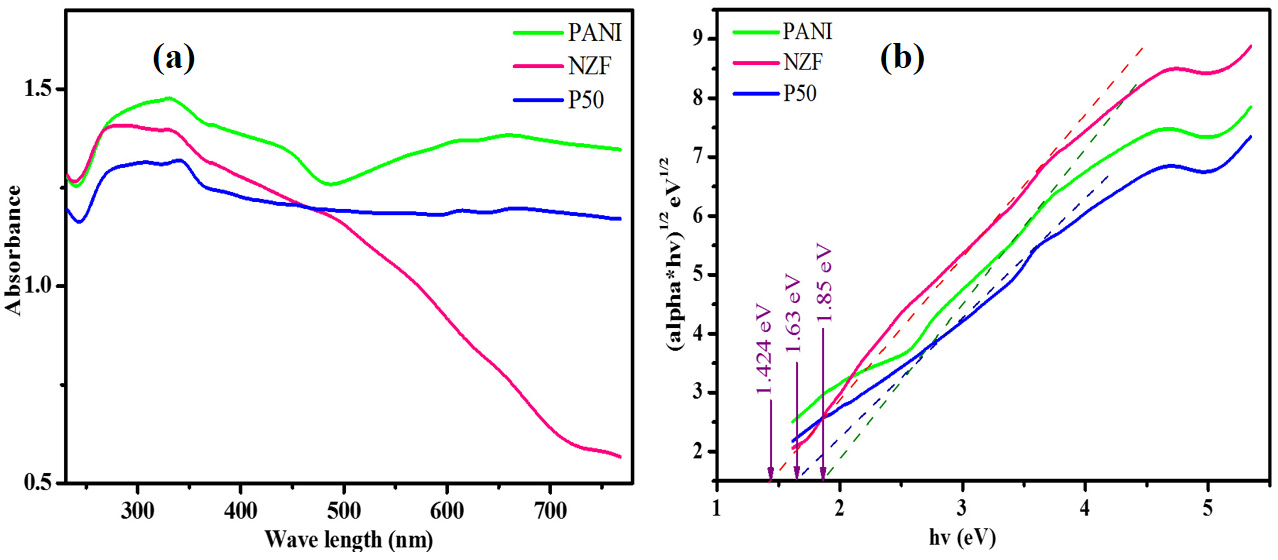
**

**Figure S7.** UV-vis diffuse relectance spectra(a) and plot of (αhυ)^1/2^ versus hυ (b) for the band gap energies of the NZF, PANi and NZF@PANi.

**Table S1.** Physical characteristics and Kinetic data of NZF and NZF@PANi.

| Composite | Average Size (nm) | Lattice spacing (Å) | Rate Constant (k, ( l/mg)^-1^ h^-1^) | R^2^ (second order) | R^2^ (pseudo first order) |
| --- | --- | --- | --- | --- | --- |
| NZF | 11 | 2.54 | 0.036 | 0.9954 | 0.9781 |
| NZF@PANi | 14.229 | 2.53 | 3.864 | 0.9989 | 0.7591 |
